# Improving diabetes disease patients classification using stacking ensemble method with PIMA and local healthcare data

**DOI:** 10.1016/j.heliyon.2024.e24536

**Published:** 2024-01-19

**Authors:** Md Shamim Reza, Ruhul Amin, Rubia Yasmin, Woomme Kulsum, Sabba Ruhi

**Affiliations:** Department of Statistics, Pabna University of Science and Technology, Pabna, 6600, Bangladesh

**Keywords:** Diabetes, Classification, Machine learning, Deep learning, Staking ensemble, Early diagnosis

## Abstract

Diabetes mellitus, a chronic metabolic disorder, continues to be a major public health issue around the world. It is estimated that one in every two diabetics is undiagnosed. Early diagnosis and management of diabetes can also prevent or delay the onset of complications. With the help of a variety of machine learning and deep learning models, stacking algorithms, and other techniques, our study's goal is to detect diseases early. In this study, we propose two stacking-based models for diabetes disease classification using a combination of the PIMA Indian diabetes dataset, simulated data, and additional data collected from a local healthcare facility. We use both the classical and deep neural network stacking ensemble methods to combine the predictions of multiple classification models and improve classification accuracy and robustness. In the evaluation protocol, we used both the train-test and cross-validation (CV) techniques to validate our proposed model. The highest accuracy is obtained by stacking ensemble with three NN architectures, resulting in an accuracy of 95.50 %, precision of 94 %, recall of 97 %, and f1-score of 96 % using 5-fold CV on simulation study. The stacked accuracy obtained from ML algorithms for the Pima Indian Diabetes dataset is 75.03 % using the train-test split protocol, while the accuracy obtained from the CV protocol is 77.10 % on the stacked model. The range of performance scores that outperformed the CV protocol 2.23 %–12 %. Our proposed method achieves a high accuracy range from 92 % to 95 %, precision, recall, and F1-score ranges from 88 % to 96 % using classical and deep neural network (NN)-based stacking method on the primary dataset. The proposed dataset and ensemble method could be useful in the early detection and treatment of diabetes, as well as in the advancement of machine learning and data analysis techniques in the healthcare industry.

## 1 Introduction

Diabetes-related diseases are a rapidly growing concern issue on the planet. Surprisingly, the prevalence of diabetes is rising rapidly worldwide. There were 424.0 million diabetics globally in 2017, and experts predict the number will rise to 628.6 million by 2045 [1]. Diabetes-related deaths increased by 0.6 million to 1.7 million worldwide between 1990 and 2017 [2]. Diabetes develops when blood sugar levels rise over standard levels. When body is unable to properly produce or use its insulin, it appears as this condition [3,4]. There are mainly two types of diabetes that are most frequently diagnosed are type 1 and type 2 [5,6]. Insulin-dependent diabetes, also known as type 1 diabetes, is a chronic autoimmune disorder in which the body's immune system attacks and destroys the pancreas's insulin-producing beta cells. At present, the most prevalent kind of diabetes is type 2, often known as non-insulin-dependent diabetes. Since type 2 diabetes commonly appears after age 35, it is also known as adult-onset diabetes. However, Type 2 diabetes is increasingly occurring in an increasing proportion of children, young people, and ethnic people [1,7]. Correct classification of diabetes can help doctors as well as patients with proper treatment planning procedures. Despite the fact that over the past few years, machine learning (ML) and deep learning (DL) algorithms have demonstrated promising results on diabetes diagnosis and classification tasks. However, when confronted with complex medical datasets, a single predictive model frequently exhibits limitations and cannot produce reproducible results. To get over these limitations, researchers have developed a variety of stacking ensemble approaches that more properly capture this issue than single traditional ML and DL algorithms [8]. Stacking is an advanced ensemble learning technique that builds a new model by combining predictions from numerous models. In stacking, several weak learners are taken into account, trained simultaneously, and then combined by training a meta-learner to make a prediction based on the predictions of the various weak learners. A meta-learner takes the predictions as features and the ground truth values as the target in data and tries to figure out how to combine the input predictions to generate a superior output prediction [9]. Recent developments in the study of ML and DL methods emphasize the need to consider stacking ensemble algorithms rather than single conventional ML and DL models because they offer more flexibility. In comparison to single ML and DL models, it increases classification accuracy, and dependability, and minimizes error [10]. We have advocated using a stacking ensemble approach in the medical field to significantly impact the early diagnosis, prediction, and preventative treatments for diabetes as part of efforts to enhance care for diabetic patients. So, we proposed a reliable methodology based on the stacking ensemble method to overcome the restriction of utilizing different single traditional ML and DL models which outperforms other classical ML and DL approaches [11]. In this paper, we attempt to provide a robust stacking ensemble framework for predicting diabetes at the onset of disease to aid health practitioners in managing the disease, decreasing its impact, and saving time and money.

Using existing conventional diabetes datasets such as the PIMA Indians diabetes dataset, numerous researchers have attempted to construct a system for the detection of diabetes using advanced ML and DL approaches [[12], [13], [14], [15]]. We go into great depth about the researcher's contribution to the PIMA diabetes dataset in the literature review section. This dataset was obtained from the Kaggle repository which is cited in the ‘Dataset Availability’ section. This dataset is comprised of 768 patients of which 268 tested positive and 500 tested negative for diabetes. ML and DL algorithms require a lot of data to train to diagnose any disease effectively and quickly. However, there is just a small amount of diabetes-related data online. On this dataset, the majority of the researchers submitted their work. We are aware that if we have big data, the machine might be more effective. In this regard, we collected diabetes data from the Pabna Diabetes Hospital (PDH) in Bangladesh. PDH is a recognized hospital that specializes in treating and monitoring diabetic patients. By training on local data, the machine learning model can be fine-tuned to capture subtle patterns or factors that are specific to the local patient population, resulting in improved diagnostic accuracy. This data collection includes 465 patients with 10 attributes, 373 people who have diabetes, and 92 people who don't. Both datasets show a significant imbalance. The negative class in the PDH data is represented four times as often as the positive class, compared to two times in the PIMA diabetes data for the negative class. Table 1 details the attributes' missing values and outliers of both datasets. However, this paper is divided into several sections the introduction is discussed in section 1, the literature review in section 2. Section 3 follows with a discussion of the proposed dataset description and methodology. In section 4, explored the experimental findings and discussion. Finally, section 5 discusses the conclusion and future work.

**Table 1:** Observed missing values and outliers for PIMA Indians and primary diabetes dataset.

PIMA Indians Diabetes Dataset			Our Primary Diabetes Dataset		
Attributes	No. of Missing Values	Observed Outliers	Attributes	No. of Missing Values	Observed Outliers
Pregnancies	111	4	Pregnancies	No	9
Glucose	5	0	Glucose Consumption	No	2
Blood Pressure	35	8	BP (Systolic)	No	4
Skin Thickness	227	4	BP (Diastolic)	No	2
Insulin	374	20	Insulin	No	5
BMI	11	5	BMI	No	5
Age	0	11	Age	No	2
Diabetes Pedigree Function	0	5	Skin Thickness(mm)	No	1
			Genetic	NO	4

## 2 Literature review

Over the last few years, different statistical techniques have been shown an important role in predictive classification for various clinical diagnosis purposes such as early diabetes diagnosis, early hypertension detection, and early cancer detection, etc. [14,[16], [17], [18], [19], [20], [21]]. Some of the machine learning techniques showed potential improvement in terms of accuracy, precision, and predictive classification problems to provide early predictions for medical professionals [[22], [23], [24]]. Additionally, deep learning techniques are extensively used in clinical diagnosis prediction [17,[25], [26], [27], [28]]. In this section, some of the research works related to previous studies, and our proposed algorithm is presented. Based on our literature reviews, it was found that the PIMA was mainly used to identify better algorithms for diabetic diagnosis. Sisodia et al. [18] performed several experiments using three different ML techniques namely decision tree, SVM, and Naive Bayes (NB) for the prediction of diabetes. The NB algorithm showed a maximum accuracy of 76.30 % compared to the other two algorithms [18]. M. Maniruzzaman et al. [14] used the gaussian process-based classification technique for comparing three algorithms namely NB, quadratic discriminant analysis, and LDA to investigate a classification algorithm for better diabetes prediction. In their studies, they claimed the highest accuracy of 81.97 %. C. Zhu et al. [29] proposed the k-means clustering and logistic regression-based algorithm for early diabetes prediction where principal component analysis played a significant role to reduce the dimension of the PIMA dataset for achieving better performance.

S. Benbelkacem et al. [30] proposed a methodology to envisage diabetes mellitus with the developed random forests method and compared it with other techniques namely support vector machine, REPTree, BFTree, C4.5, and SimpleCart. The results showed an efficient improvement in terms of prediction accuracy of 78 % in the case of the random forest method using 5-fold cross-validation. In another study, H. Lai et al. [12] investigated to identification of the diabetes mellitus algorithm based on the LR and Gradient Boosting (GB) machine techniques. The models were performed on only rows with non-missing values of 392 instances of the PIMA dataset where 80 % data was used as a training dataset and the remaining 20 % as a testing dataset, and also accomplished by 10-fold cross-validation. The highest accuracy achieved was 84.7 % for the logistic regression and 88 % for the GB machine technique. N. Pradhan et al. [15] presented an artificial neural network-based algorithm for predictive classification. They used 76 % of 1004 instances of data for testing and the remaining 24 % for testing model validation. The accuracy was attained by 87 %. Wu. H. et al. addressed PIMA dataset for predicting type-2 diabetes mellitus [16]. In their research, they used 10-fold cross-validation to authenticate the performance of the better K-means algorithm and the logistic regression algorithm. S. S. Bhat et al. [31] analyze a genuine clinical dataset on diabetes disease, acquired from a doctor in the Indian area of Bandipora between April 2021 and February 2022. In their experimental investigation, six machine learning algorithms were effectively applied. Random Forest is the most accurate classifier, with a 98 % accuracy rate. They balance the data set using four data balancing techniques: random under-sampling, random over-sampling, Tomek-links, and SMOTE. H. B. Kibria et al. [32] proposed a machine-learning strategy based on explainable AI to identify diabetes. They test their suggested strategy in diabetes prediction using the well-known Pima Indian Diabetes dataset. To handle the dataset, they employ the median to impute missing values and SMOTE to balance it. An ensemble classifier is used in conjunction with six learning algorithms. Their suggested weighted ensemble model achieves 90 % accuracy. In another study, S. S. Bhat et al. [33] attempted to develop a model with several variables based on the PIMA dataset, to analyze diabetic patients and diabetes diagnosis using various machine learning techniques. They use the train-test method to split the dataset, 80 % of the data in the dataset is used for training, whereas 20 % is used for testing. Correlation feature selection evaluates performance by selecting relevant features. According to the testing results, Random Forest outperformed the other two machine learning approaches in terms of accuracy (97.75 %). Patil et al. [34] advocated a stacking-based ensemble employing a non-dominated sorting genetic algorithm (NSGA-II) scheme. The NSGA-II was used for feature selection, and a stacking technique was used to predict Type 2 diabetes. Stacking uses the classifying skills of several high-performing models to provide better results than any one model in an ensemble. On the Pima diabetes dataset, the NSGA-II with stacking model has an accuracy of 81.9 %. The gathered dataset was also used in this experiment, and the NSGA-II with stacking model predicts diabetes with an accuracy of 88.18 %. S. S. Bhat et al. [35] developed a hybrid model to predict type 2 diabetes patients (T2DM) using three ML models including logistic regression, decision tree, and random forest algorithm. The logistic regression model achieved an accuracy of 99.34 %, marking the highest prediction rate recorded. This study also highlights the significant risk factors for developing the risk of diabetes.

The prediction accuracy was 95.42 % compared to the 92.38 % accuracy of the hybrid prediction model proposed by Patil et al. [36]. In their data preprocessing step, 192 instances were deleted because of incorrect classification made by K-means clustering. The remaining instances of 625 were used in the C4.5 algorithm with a 10-fold cross-validation method. M. F. Ijaz et al. [37] proposed a hybrid model for hypertension and type-2 diabetes prediction. In the preprocessing and data impute techniques, outlier data was removed utilizing the density-based spatial clustering of applications with noise-based technique and the SMOTE technique used for class balance, the model accuracy improved by their proposed prediction model is 92.55 %. The concluding remarks of the literature review reveal that the accuracy of the various algorithms for the same PIMA dataset heavily depends on data preprocessing and impute techniques. Some researchers claimed better algorithms for the early diabetics’ diagnosis model. However, the results have been limited to the role of missing values, class imbalance, and overfitting reduction. To improve patient classification accuracy, we propose in this paper a deep neural network stacking approach for the classification of data due to diabetes mellitus.

## 3 Materials and methodology

We conduct a rigorous investigation into diabetes classification using different statistical data preprocessing techniques that optimizes the data for machine learning algorithms, making them more effective in capturing patterns and relationships within the data. For developing predictive model, stacking is used to leverage the strengths of different models by blending their individual predictions, thereby creating a more powerful and accurate ensemble model. The proposed methodological framework describes in the following subsection. Details dataset preprocessing and proposed methodology are shown in Fig. 2.

### 3.1 Dataset description

We employed three separate datasets in our investigation. The primary goal of the research is to create a predictive model based on ML and DL to accurately classify diabetes patients. In this study, we also collected new benchmark diabetic information to verify our assessment and the information was collected from the Pabna Diabetes Hospital, Pabna, Bangladesh. Local healthcare data may serve a more diverse population compared to publicly available datasets. The data is collected retrospectively for our study. To address anonymity concerns, all collected data underwent a rigorous anonymization process to ensure the confidentiality and privacy of individuals involved. Every individual who took part in this research gave their informed consent before beginning. Following the ethical guidelines provided by the Pabna Diabetes Hospital, Pabna-6600, Bangladesh, the informed consent procedure was carried out (Ref No.: CERT/PADAS/PAB-64). The study's protocols, potential risks, benefits, and aims were all thoroughly explained to the participants. This diversity can help in training ML models that are robust and generalize well to different patient groups. There are 465 female patients in this dataset that are at least 21 years old, comprising 373 diabetic patients and 92 non-diabetic patients, 131 patients with serum insulin and 334 patients without any serum insulin level, 293 patients who inherited it genetically, and 172 patients who did not have it genetically. We partitioned the data set into two parts with nine different risk variables (number of pregnancies, age, BMI, systolic and diastolic blood pressures, number of genetic, serum insulin, triceps skinfold thickness, plasma glucose concentration (in an oral glucose tolerance test after 2 h) and the other with diabetic and non-diabetic.

Furthermore, we consider the PIMA dataset along with its relevance for diabetes research and its accessibility for ML model evaluation. This dataset was collected from the Kaggle repository, a renowned open-source website for researchers. The primary source of this dataset was the National Institute of Diabetes and Digestive and Kidney Diseases in India, where all of the patients are of Indian descent. This dataset contained 768 uniquely recognized observations, 268 of which were positive for diabetes (encoded as 1) and 500 of which were negative for diabetes (encoded as 0) and there are nine columns with eight independent features.

**Fig. 1:**
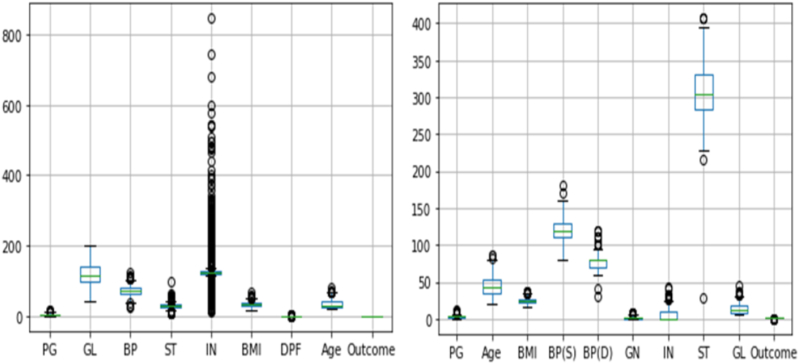
Box plot for PIMA (left) and our primary diabetes dataset (right).

To generalize and strengthen proposed predictive approaches we generated a synthetic dataset. 1000 samples were constructed with ten characteristics, seven of which were informative and three of which were redundant, and the target dataset's class labels were set to two. Table 1 shows the demographic and clinical profile of both dataset in details. However, in the PIMA dataset, 376 out of 768 occurrences lacked experimental validity because zero values were reported in place of missing experimental observations for a few attributes. The zero values found in qualities like pregnancy, blood pressure, skin thickness, insulin, and BMI are not biologically conceivable. These zero values considered as missing values. Our primary dataset does not contain any missing values. Both the PIMA dataset and primary dataset contain outliers or data values that are unusually extreme. The proposed primary and the PIMA dataset outliers are displayed in Fig. 1.

**Fig. 2:**
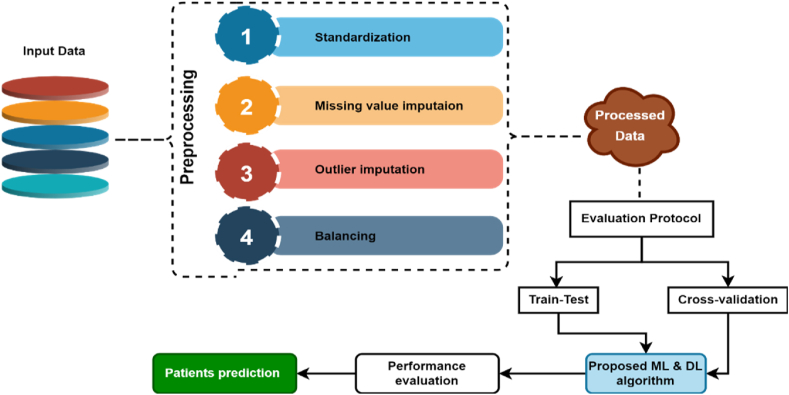
An overview of the proposed research approach workflow.

### 3.2 Preprocessing

Processing is the task that cleans the noisy, messy, erroneous observations from the data set. Before feeding any algorithm, treatment of missing values, outliers detection, scaling, and balancing are required to ensure data quality [38,39], [40,41]. In this study, the z-score, also known as a standard score, is used to determine how much the data points deviate from the mean. Subsequently, we employed the interquartile range (IQR) approach to look for outliers that could have an impact on the prediction. Using the IQR to identify outliers and the box plot to visually confirm their presence, we then consistently replaced these outlier values with the median. Additionally, the median imputation approach is utilized to fill in the missing values to ensure a robust analysis and reduce the impact of extreme values. SMOTE has been used to balance the class skewedness.

### 3.3 Proposed ML and DL based stacking implementation strategy

**Fig. 3:**
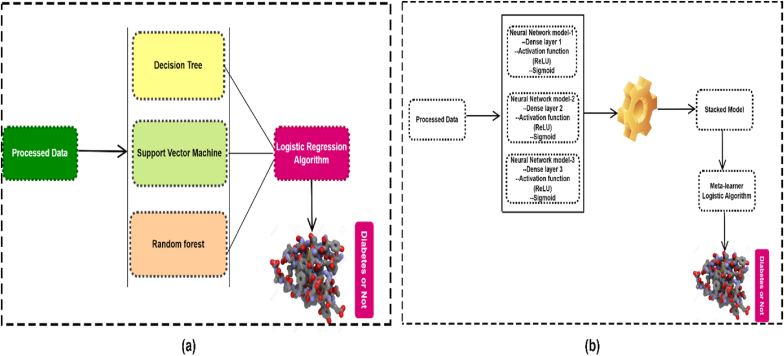
Proposed ML (a) and DL (b) staking ensemble model architecture.

In the proposed method, three customized neural networks and three ML algorithms are considered for stacking ensemble. In the ML based hybrid staking approach, Decision Tree (DT), Random Forest (RF), and Support Vector Machine (SVM) are developed as level-0 models, and logistic regression is used as a meta-learner. As a base model, the DT fit to the training set, and the prediction is made on the testing dataset. Similarly, on the training dataset, RF and SVM were fit, respectively, as base models, and prediction is generated on the testing dataset. The prediction from the testing set from three ML model are considered as new features to train the meta-model. Finally, the results obtained from meta learner model on test dataset. Fig. 3 (a) shows in details.

In the neural network approach, three customized sequential model is developed as level-0 model, and logistic regression is used as a meta learner. A dense layer with a specific number of neurons is created, and depending on the number of features, the requisite number of dimensions in the input layer is expected. To avoid the vanishing gradient problem, the rectified linear unit (ReLU) activation function f(x)=max(0,x) is utilized. If the function received any negative of x then the function returned 0 value otherwise it stores the actual value for any value of x since this is a binary classification problem, one neuron was placed in the output layer with a sigmoid activation function. The sigmoid function $S\left(x\right)=\frac{1}{1+e^{-x}}$ classified our patients by using a 0 to 1 code. If the probability of the disease is getting large then it encoded disease exits otherwise not. The loss function is estimated using binary cross entropy, which is a highly convenient way to train a model to solve a classification issue. The Adam optimizer is used and the number of epochs is set up by 200. The details NN model architecture shows in Table 2, and Fig. 3 (b). In this stacking experimental setup, a base neural network is generated and fit to the training set, with a dense layer of 25 neurons, 9 neurons placed in the input layer according to the number of features, and the prediction is recorded as well as the model was stored.

**Table 2:** Proposed three customized neural network model architecture for stacking.

Neural Network model-1	Neural Network model-2	Neural Network model-3
Input Dimension = 9 Dense Layer = 12 Activation Function = ReLU Dense Layer = 1 Activation Function = Sigmoid	Input Dimension = 9 Dense Layer = 12 Activation Function = ReLU Dropout = 0.25 Dense Layer = 8 Activation Function = ReLU Dense Layer = 1 Activation Function = Sigmoid	Input Dimension = 9 Dense Layer = 10 Activation Function = ReLU Dense Layer = 6 Activation Function = ReLU Dense Layer = 8 Dropout = 0.25 Dense Layer = 1 Activation Function = Sigmoid

Then on the training dataset, three neural networks with different architectures were developed. To avoid overfitting the problem dropout layer was taken with 0.25 ratio predictions recorded, and the models were stored. Since there were three neural networks in the test set, 300 samples resulted in four arrays with the shape [300, 1], which were then combined into a three-dimensional array with the shape [300, 3, 1]. 300 samples with a certain number of features were required as input for a new model; given that three models were utilized, each with one prediction in each example, the sub-models were given three (1*3) features for each example. The sub-model predictions were transformed into a [300, 3] shaped array, which is then used to train a meta-learner. The dataset for training the meta-learner is then constructed, the meta-learner is fit to the data for training and a final prediction is made.

## 4 Experimental results and discussion

In this section, we present the results of our experiments aimed at classifying diabetes subtypes using proposed two stacking approaches. The dataset used for our experiments consists of simulated, PIMA and clinical data collected from local healthcare system. To validate our proposed model, we used both the train-test and cross-validation (CV) techniques in the evaluation protocol. For our empirical study, we divided 70 % into training set, 30 % for the testing set, and applied a 5-fold cv to all three datasets (PIMA, Primary, Simulation).

### 4.1 Evaluation protocols

Several assessment matrices, including accuracy, precision, recall, and f-1 score, were taken into consideration while trying to classify diabetes patients correctly. To classify a single diabetes patient four different outcomes are considered such as false positive (FP), false negative (FN), true positive (TP), and true negative (TN). In contrast to false negatives, which are the opposite of false positives, false positives suggest that people have diabetes when they do not.

$$Accuracy=\frac{TP+TN}{TP+TN+FP+FN}\;;\;Precision=\frac{TP}{TP+FP};\;Recall=\frac{TP}{TP+FN}\;;\;F1\;Score=2*\frac{precision*recall}{precision+recall}$$

### 4.2 Results discussion

On the simulated data, ML algorithms like Decision Tree, Random Forest, and SVM have been developed, and 89.01 % stacked accuracy was achieved by fitting a stacked model with meta learner logistic regression. Following that, three alternative NN architectures were used, and 93.70 % stacked accuracy is reached by fitting the stacked model with meta learner logistic regression. The proposed stacked NN model achieved the best accuracy of 95.50 % in the CV protocol on this dataset as well and other evaluation matrices performed well ranging from 1 to 2% in terms of precision, recall, and f1 score shows in Table 3. Moreover, the highest accuracy is achieved by stacking Ensemble with three NN architectures, which results in an accuracy of 95.50 %, precision of 94 %, recall of 97 %, and f1-score of 96 % using 5-fold cv.

**Table 3:** Performances (%) of ML and NN algorithms on a simulated dataset using train-test and CV.

Protocol	Algorithm	Accuracy	Precision	Recall	F1-Score
Train-Test	DT	81.33	.81	.81	.81
	RF	88.33	.88	.88	.88
	SVM	93.33	.93	.93	.93
	Staking Ensemble	89.01	.89	.89	.89
	DNN1	93.32	.93	.93	.93
	DNN2	92.05	.92	.92	.92
	DNN3	91.02	.91	.91	.91
	Staking Ensemble	93.70	.95	.94	.94
Cross-validation	DT	83.40	.84	.83	.83
	RF	89.59	.89	.90	.89
	SVM	91.40	.90	.92	.91
	Staking Ensemble	91.90	.90	.92	.91
	DNN1	94.00	.92	.96	.94
	DNN2	93.50	.91	.96	.94
	DNN3	94.50	.93	.96	.95
	Staking Ensemble	95.50	.94	.97	.96

Table 4 shows the outcomes of ML techniques and NN, respectively, using the PIMA dataset. For the Pima Indian Diabetes dataset, the stacked accuracy acquired from ML algorithms is 75.03 % using the train-test split protocol, while the accuracy gained from the CV protocol is 77.10 % on the stacked model. RF classifier gives the highest 79.33 % accuracy, 80 % precision, 79 % recall, and 79 % f1-score among all ML and neural network models. The performance score that outperformed the CV protocol ranged from 2.23 % to 12 %. Table 5 presents the outcomes of ML techniques and neural networks, respectively, using our primary diabetes dataset.

**Table 4:** Performances (%) of ML and NN algorithm on pima dataset using train-test and CV.

Protocol	Algorithm	Accuracy	Precision	Recall	F1-Score
Train-Test	DT	65.08	.65	.65	.65
	RF	79.33	.80	.79	.79
	SVM	69.03	.69	.69	.69
	Staking Ensemble	75.03	.75	.75	.75
	DNN1	68.80	.64	.74	.69
	DNN2	64.15	.64	.75	.68
	DNN3	65.40	.68	.69	.67
	Staking Ensemble	68.05	.68	.68	.62
Cross-validation	DT	68.31	.65	.68	.67
	RF	76.81	.77	.79	.78
	SVM	68.61	.68	.70	.69
	Staking Ensemble	77.10	.68	.70	.69
	DNN1	63.50	.63	.66	.64
	DNN2	64.50	.64	.65	.65
	DNN3	63.50	.64	.62	.63
	Staking Ensemble	65.50	.66	.65	.65

**Table 5:** Performances (%) of ML and neural network algorithms on primary dataset using train-teat and CV.

Protocol	Algorithm	Accuracy	Precision	Recall	F1-Score
Train-test	DT	95.98	.96	.95	.96
	RF	95.98	.94	.96	.96
	SVM	90.18	.91	.90	.90
	Staking Ensemble	95.08	.95	.95	.95
	DNN1	88.40	.89	.88	.88
	DNN2	90.62	.91	.91	.91
	DNN3	90.20	.91	.90	.90
	Staking Ensemble	92.04	.92	.90	.92
Cross-validation	DT	96.91	.97	.96	.96
	RF	96.37	.98	.95	.96
	SVM	88.17	.96	.79	.87
	Staking Ensemble	96.64	.96	.79	.87
	DNN1	87.20	.85	.91	.88
	DNN2	90.32	.91	.96	.93
	DNN3	94.60	.92	.97	.95
	Staking Ensemble	94.60	.92	.97	.95

**Fig. 4:**
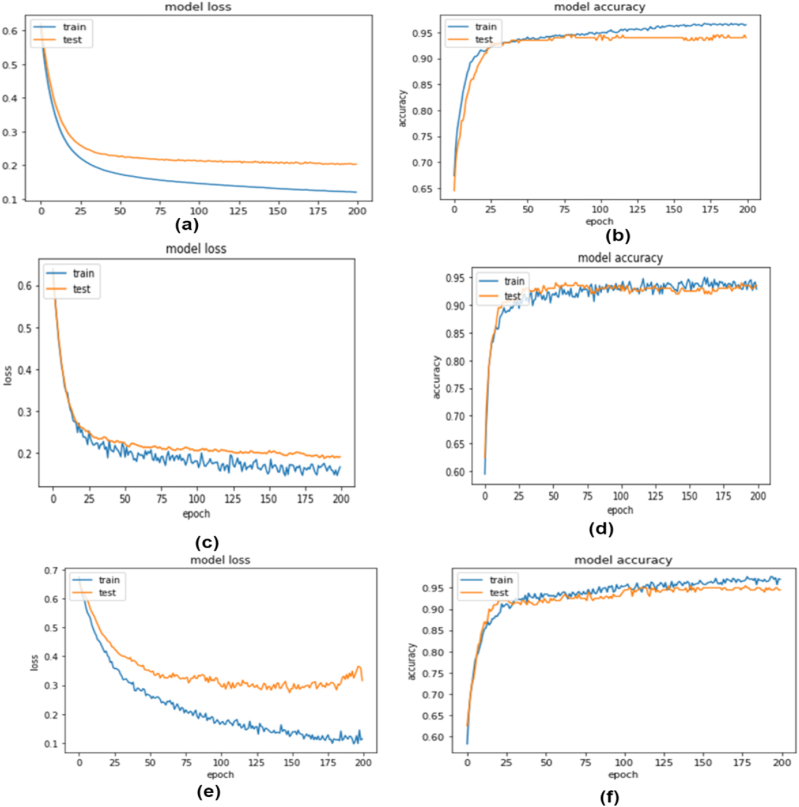
Loss & Accuracy curve obtained from NN model1 (a, b), model2 (c, d) model3 (e, f) for simulation data.

**Fig. 5:**
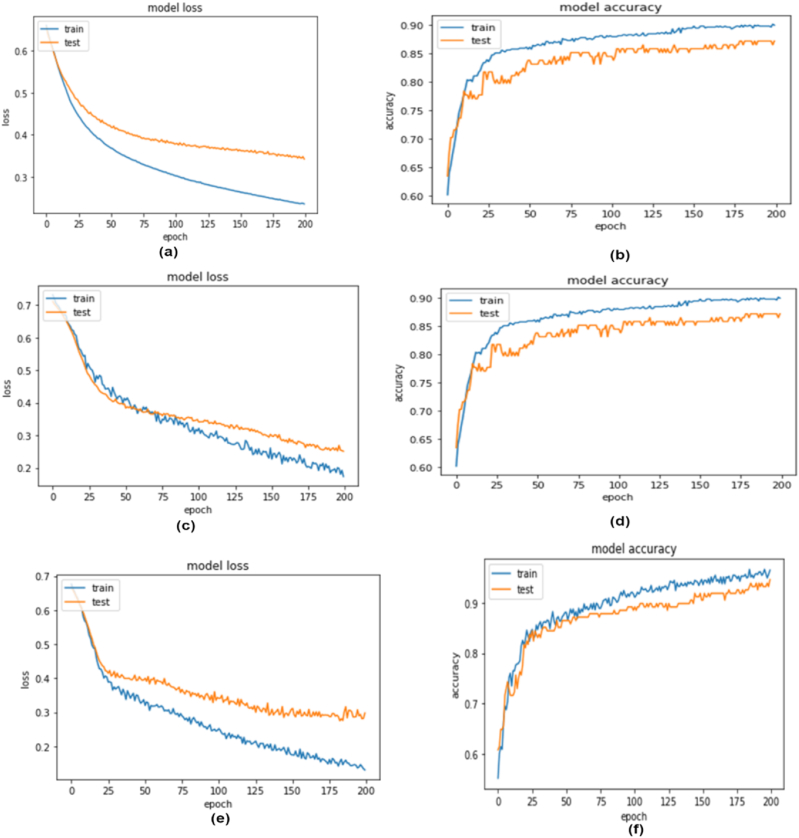
Loss & Accuracy curve obtained from NN model1 (a, b), model2 (c,d) model3 (e,f) on primary dataset.

The DT classifiers perform best on the cross-validation technique, with an accuracy of 96.61 %, precision of 97 %, recall of 96 %, and f1 score of 96 %. The stacked ensemble accuracy obtained from ML algorithms in our primary diabetes dataset is 96.64 %, whereas the accuracy obtained with three stacked neural networks is 94.60 % with 92 %

precision, 97 % recall, and 95 % f1-score. Despite not having the best performance results, the stacking model's results on primary and outer performance on simulation data are robust and support the validity of our proposed models, according to the prediction performance analysis. Fig. 4 depicts the performance curves for three distinct NN models. Subfigures (a) and (b) illustrate the loss and accuracy curves for the first NN model, while (c) and (d) showcase the corresponding curves for the second NN model. Finally, subfigures (e) and (f) shows the loss and accuracy curves for the third NN model. A snapshot of the training process and the direction in which the NN learns incrementally is provided by the accuracy and loss curves. Based on how well the model performs in the training and test sets, the loss is determined. It is the total of all mishaps produced during training or testing sets for each epoch. A model's loss value indicates how effectively or how cheaply it performs after each optimization cycle. A model's accuracy score serves as a metric of how well the prediction made by the fitted model reflects the actual data. In Fig. 4, the model2 (c, and d) training and testing performance is closer to the other two models, and the loss curve is also closed. The training accuracy shows that it achieved 96.6 % whereas testing accuracy is approximately the same here. Fig. 5 depicts the performance curves for three distinct NN models. Subfigures (a) and (b) illustrate the loss and accuracy curves for the first NN model, while (c) and (d) showcase the corresponding curves for the second NN model. Finally, subfigures (e) and (f) shows the loss and accuracy curves for the third NN model. Although this model performs slightly worse than model 3 in terms of performance, the accuracy curve and model loss curve for NN model 2 among these three models show that they achieved training accuracy of 94.3 % and testing accuracy of 94.6 %. Due to the smaller widening gap between the training and testing loss curves, this model is also better suited to combat the overfitting issue than model 3.

### 4.3 Performance comparison with the similar existing study

Innumerable researchers have attempted to predict diabetes on several diabetes data using different methods, some of which we included in the literature review section. The fundamental strategy is to develop a model that can accurately predict the onset of diabetes mellitus. A. Naseem et al. [42] developed a hybrid strategy that combines an IoT and deep learning model to effectively detect diabetes. The applied six classifiers were SVM, logistic regression, ANN, CNN, RNN, and LSTM, using four metrics: accuracy, F1-score, recall, and precision. RNN classifier outperformed all other classifiers with an accuracy of 81 %, precision of 75 %, recall of 49 %, and F1-score of 65 %. C. Y. Chou et al. [43] proposed Microsoft Machine Learning Studio to train the models of various kinds of neural networks on 15,000 women patients aged between 20 and 80 years. Among the all-predictive model, the two-class boosted decision tree achieved an accuracy of 0.953 %, and other evaluation scores ranged from 62.90 % to 92.10 %. H. Chu et al. [44] proposed an automated deep-learning model to determine how psychological risk factors like anxiety and sadness affect the likelihood that people with T2DM may develop cardiovascular diseases. This is a cross-sectional study using a single dataset and also a small number of patients (834), which does not justify a causal inference for the deep learning model. Accuracy scores for the DL model reached 87.50 %, sensitivity of 88.06 %, and specificity of 87.23 %, respectively. Tasin et al. [45] used a Bangladeshi female patients dataset. In their proposed approach, they used mutual information feature selection techniques, SMOTE and ADASYN for data balancing, and several ML algorithms to develop the predictive model for diabetes patients’ prediction. The XGBoost classifier has the highest accuracy (81 %) when using the ADASYN technique. Hasan et al. [46] proposed a comprehensive framework, including validation, scaling, missing value imputation, and outlier rejection. An AUC value of 0.95 % was attained by their proposed models. However, in our proposed dataset the ML model performed better than the NN model, it achieved an accuracy of 96.91 while the stacking ensemble of the NN model validated the performance for the simulation data giving an accuracy score of 95.50 %. Table 6 shows several performance comparisons against our proposed approach.

**Table 6:** State-of-the-art result comparison of diabetes disease classification.

Author	Year	Accuracy (%)
A. Naseem et al. [42]	2022	81
C. Y. Chou et al. [43]	2023	95.30
H. Chu et al. [44]	2021	87.50
Tasin et al. [45]	2023	81
Hasan et al. [46]	2020	×
A. Mujumdar et al. [47]	2019	96
Sonar et al. [48]	2019	×
Rajendra et al. [49]	2021	93
Ramesh et al. [50] Proposed study	2021 2023	83.20 96.91

### 4.4 Strength, limitation, and extension

This paper intended to predict diabetes patients accurately using both ML and DNN approach. We utilized robust statistical techniques to ensure the best data quality, including deterring information loss, reducing noise, and reducing bias. These techniques included imputing missing values, anomalies by median value, scaling the dataset by standardized, and making the dataset's unique features scale and model more effectively. To ensure stability and capture the complicated pattern of the data, we built both ML and DNN models for the proposed model. Both models were validated using the train-test and CV approaches. In the designed phase, the logistic regression is employed as a meta learner to avoid overfitting, permit L1, L2 regularization, etc. Moreover, we simulated a dataset that demonstrated the model's effectiveness in order to strengthen the study. However, utilizing the proposed approach, we were able to accomplish classification rates of 95.50 %, 77.10 %, and 96.91 % on the simulated, PIMA, and primary dataset, respectively. This is the highest of several recent studies that were comparable to ours. Despite its strong performance, this study nevertheless has inherent weaknesses. The major concern is that there aren't large amount of features and observations, which is worrisome because more relevant dominating risk features are needed to extract useful information utilizing feature engineering that can identify the underlying structure of the data. To function effectively, the ML model, in particular the DNN model, needs a lot of data. Additionally, in the extension, we ought to generate more clinical and genetic biomarker features (i.e., proteomics, and metabolomics data) and use data harmonization techniques with the current features to build metadata that is compatible and consistent. Following that, it is possible to apply a lot of CNN pre-trained models to accurately and successfully classify the diabetic patients. To help patients achieve and maintain optimal glucose control, build models that suggest customized dietary changes, explore telemedicine modes and remote monitoring technology operate enable healthcare providers to remotely, and pave the road for precision medicine in diabetes management.

## 5 Conclusion and future work

This study proposed two stacking ensemble methods and introduced a diabetes dataset from a local healthcare facility. In the analysis of relevant diabetes research, benchmark PIMA diabetes data is used, and a simulation study is performed to robustify the predictive model performances. We used both the train test and cross-validation techniques with different evaluation matrices to validate the proposed model. On simulated data, PIMA data, and primary data, we achieved recognition rates of 95.50 %, 77.10 %, and 96.91 %, respectively. The best recognition rate was 96.91 %, outperforming several cutting-edge classification methods. A thorough investigation into various statistical data preprocessing techniques, robust data imputation methods, combining multiple models via stacking, and additional diverse conditioned diabetes data aids in improving recognition performance. The proposed approach has significant implications for diabetes early detection and management, which can lead to improved healthcare outcomes and cost savings. Although the developed meta-ensemble approaches produced better results, they are occasionally more computationally expensive than individual models on large databases.

## Funding

This research did not receive any specific grant from any funding agency in the public, commercial or not-for-profit sector.

## Ethics declaration

A strong commitment to ethical standards and attention to pertinent guidelines characterized this study, which involved the analysis of data on human behavior. For our local healthcare dataset, the retrospective data used in our study is fully anonymized and the informed consent process was conducted in accordance with the ethical standards outlined by the Pabna Diabetes Hospital, Pabna-6600, Bangladesh (Ref. No.: CERT/PADAS/PAB-64). The study's protocols, possible risks, benefits, and aims were all thoroughly explained to the participants. The opportunity to ask questions was provided, and they were reassured that their participation is entirely optional. In order to safeguard the participants' rights and confidentiality, consent forms were acquired and recorded in accordance with ethical principles. Additionally, PIMA, which is collected from the Kaggle repository, is a well-known secondary dataset. So, ethical approval is not required for this dataset.

### Dataset availability statement

Enthusiasts can explore our primary collected dataset at this URL (https://github.com/ruhul256/Pabna-Diabetes-Dataset-Bangladesh).

For PIMA Indians diabetes dataset visit this URL (https://www.kaggle.com/datasets/uciml/pima-indians-diabetes-database).

## CRediT authorship contribution statement

**Md Shamim Reza:** Writing – original draft, Formal analysis, Conceptualization. **Ruhul Amin:** Formal analysis, Data curation. **Rubia Yasmin:** Writing – review & editing, Visualization. **Woomme Kulsum:** Writing – review & editing, Validation, Investigation. **Sabba Ruhi:** Writing – review & editing, Supervision, Investigation, Conceptualization.

## Declaration of competing interest

The authors declare that they have no known competing financial interests or personal relationships that could have appeared to influence the work reported in this paper.
